# Inhibition of DNA Methylation Impairs Synaptic Plasticity during an Early Time Window in Rats

**DOI:** 10.1155/2016/4783836

**Published:** 2016-07-14

**Authors:** Pablo Muñoz, Carolina Estay, Paula Díaz, Claudio Elgueta, Álvaro O. Ardiles, Pablo A. Lizana

**Affiliations:** ^1^Department of Pathology and Physiology, School of Medicine, Faculty of Medicine, University of Valparaíso, 2341386 Valparaíso, Chile; ^2^Interdisciplinary Center for Innovation in Health (CIIS), University of Valparaíso, 8380492 Valparaíso, Chile; ^3^Institute of Physiology I, Systemic and Cellular Neuroscience, Albert-Ludwigs University Freiburg, 79104 Freiburg im Breisgau, Germany; ^4^CINV-Universidad de Valparaíso, 2360102 Valparaíso, Chile; ^5^Instituto de Biología, Pontificia Universidad Católica de Valparaíso, 3100000 Valparaíso, Chile

## Abstract

Although the importance of DNA methylation-dependent gene expression to neuronal plasticity is well established, the dynamics of methylation and demethylation during the induction and expression of synaptic plasticity have not been explored. Here, we combined electrophysiological, pharmacological, molecular, and immunohistochemical approaches to examine the contribution of DNA methylation and the phosphorylation of Methyl-CpG-binding protein 2 (MeCP2) to synaptic plasticity. We found that, at twenty minutes after theta burst stimulation (TBS), the DNA methylation inhibitor 5-aza-2-deoxycytidine (5AZA) impaired hippocampal long-term potentiation (LTP). Surprisingly, after two hours of TBS, when LTP had become a transcription-dependent process, 5AZA treatment had no effect. By comparing these results to those in naive slices, we found that, at two hours after TBS, an intergenic region of the RLN gene was hypomethylated and that the phosphorylation of residue S80 of MeCP2 was decreased, while the phosphorylation of residue S421 was increased. As expected, 5AZA affected only the methylation of the RLN gene and exerted no effect on MeCP2 phosphorylation patterns. In summary, our data suggest that tetanic stimulation induces critical changes in synaptic plasticity that affects both DNA methylation and the phosphorylation of MeCP2. These data also suggest that early alterations in DNA methylation are sufficient to impair the full expression of LTP.

## 1. Introduction

Precise control of gene expression is essential for proper neuronal function and the integrity of the central nervous system [1]. Although several concerted mechanisms work together to control gene transcription [2, 3], DNA methylation has drawn special interest as a cellular mechanism that is capable of adapting gene expression to environmental conditions [4]. Several studies have already established the importance of DNA methylation both during development [5] and in adult animals, with a particularly emphasis on its involvement in learning processes and long-term potentiation (LTP) [6, 7]. However, little is known regarding the mechanisms that regulate DNA methylation and demethylation. This is particularly important in the adult nervous system, where the regulation of transcription can be quite dynamic and require rigorous temporal control [8, 9].

In mammalian genomes, including that of humans, the addition of a methyl group occurs exclusively at a position 5 of the cytosine, located immediately before a guanosine (CpG). An interesting fact is that only neurons, virtually absent in other cell types [10], exhibit multiple CpH methylation sites, where H corresponds to another nucleotides, in a different context to the classical CpG dinucleotide [11]. Fetal brain exhibits very low levels of CpH, which gradually increase with age [12].

Although the vast majority of CpGs in the mammalian genome are normally methylated and part of condensed chromatin [5], the regulation of gene expression through methylation/demethylation actively occurs at particular genomic regions that are enriched in sparsely methylated CpGs motifs that are known as CpGs islands [13]. The process of DNA methylation occurs through an enzymatic reaction that is catalyzed by the superfamily of DNA methyltransferases (DNMTs). These enzymes transfer a methyl group from S-adenosylmethionine (SAM) [14, 15] to a cytosine, resulting in the formation of 5-methylcytosine (5mC). DNMT-3A and 3B catalyze de novo methylation, while DNMT1 is responsible for the maintenance of previously methylated sites in the adult brain [16].

Interestingly, DNMT1 is highly expressed in postmitotic neurons [17], suggesting an alternative role for DNMT1. On the other hand, a recent study showed that azanucleosides inhibitors (5AZA) could induce DNA damage [18], thus recruiting repair machinery and DNMT1 to double-strand cleavage sites [19], which could explain why these inhibitors can demethylate even in the absence of cell division.

In contrast to DNA methylation, the mechanism underlying demethylation involves the DNA-repair system protein GADD45 and a family of proteins that includes oxygenase TETl, which oxidize 5mC to 5-hydroxymethyl, 5-formyl, or 5-carboxyl cytosine [8, 20]. However, the precise role of these intermediaries remains unknown.

One of the main effectors of DNA methylation-dependent gene regulation is methyl-CpG-binding protein 2 (MeCP2) [21], a transcriptional factor that reads the methylation of several genes and controls their expression by recruiting corepressors to their promotor region [22]. The MeCP2 gene is strongly expressed in the brain, and mutations in MeCP2 have been associated with delayed neuronal maturation and neuropsychiatric disorders, including Rett syndrome [23]. In turn, MeCP2 is dynamically regulated by neuronal activity mainly via the differential phosphorylation of key residues that modulate its affinity to its partners, which affects downstream gene expression and cellular responses to environmental variation [3, 24, 25].

Few studies have explored the involvement of DNA methylation and MeCP2 modifications during the different temporal stages of processes that involve active gene regulation, such as synaptic plasticity [26]. Here, we approach this question by studying changes in the methylation of the reelin (RLN) gene. This gene encodes an extracellular matrix protein that contacts postsynaptic dendritic spines via the very low-density protein receptor (VLDLR) and the apolipoprotein E receptor 2 (ApoER2). In the adult brain, RLN is secreted by GABAergic interneurons and is critical for synaptic plasticity and memory formation [27, 28]. Several reports have suggested that the RLN gene may be acutely regulated by DNA methylation [29, 30] and changes in the binding of MeCP2 to the RLN promoter [31]. In acute hippocampal slices obtained from rats, the inhibition of DNA methylation using azanucleosides inhibitors affected both the induction and the expression of Schaffer collateral-CA1 pyramidal cell LTP that was induced using high frequency stimulation [7]. We investigated the time window during which LTP is sensitive to azanucleosides inhibitors and the correlated dynamic changes in MeCP2 phosphorylation and the methylation state of RLN.

These results help us better understand the role of DNA methylation in synaptic plasticity.

## 2. Materials and Methods

### 2.1. Subjects

Male Sprague Dawley rats (21 days old) were obtained from the animal facility of the University of Valparaíso. They were housed under standard conditions at a constant temperature and with a 12-hour light/dark cycle with food and water provided ad libitum. All experiments were performed in accordance with the guidelines of the Bioethics Committee of the University of Valparaíso for Animal Research for the treatment and care of animals.

### 2.2. Electrophysiology

Hippocampal slices (400 *μ*m) were cut from rat brain tissues in ice-cold dissection buffer (in mM: 212.7 sucrose, 2.6 KCl, 1.23 NaH2PO4, 26 NaHCO3, 10 dextrose, 3 MgCl2, and 1 CaCl2, bubbled with a mixture of 5% CO2 and 95% O2). Slices were incubated for 1 h at room temperature in Artificial Cerebrospinal Fluid (ACSF, in mM: 124 NaCl, 5 KCl, 1.25 NaH2PO4, 26 NaHCO3, 10 dextrose, 1.5 MgCl2, and 2.5 CaCl2, continuously equilibrated with 5% CO2 and 95% O2). Synaptic responses were evoked by stimulating the Schaffer collaterals using concentric bipolar stimulating electrodes (0.2 ms), and field excitatory postsynaptic potentials (fEPSPs) were recorded using extracellular electrodes that were filled with ACSF and placed in the CA1* stratum radiatum* [32, 33]. Control responses were recorded using half-maximum stimulation intensity at a frequency of 0.033 Hz. LTP was induced using a theta burst stimulation (TBS) protocol. The stimulation consisted of four theta epochs that were delivered every 10 s. Each epoch consisted of 10 trains of four pulses at 100 Hz that were generated at a frequency of 0.5 Hz [32, 33]. When testing the effect of a pharmacological agent, recordings were made using slices from the same animal in two independent submersion-recording chambers (32 ± 0.5°C), one of which was superfused with vehicle-containing ACST, while the other was superfused with drug-containing ACSF. All data are presented as mean ± SEM.

### 2.3. Pharmacological Stimulation of Hippocampal Slices

Hippocampal slices were stabilized in oxygenated ACSF (32°C) for 1 h and then incubated for 60 min with vehicle (0.001% DMSO), actinomycin-D (25 *μ*M), to block transcription and vehicle (0.001% CH3COOH) or 5-aza-2-deoxycytidine (5AZA, 30 *μ*M) to inhibit DNA methylation. The hippocampal CA1 area was subsequently microdissected for the DNA methylation assays.

### 2.4. DNA Methylation Assay

Genomic DNA was isolated from hippocampal CA1 microdissected tissues using a Wizard genomic DNA purification kit (Promega, Madison, WI) according to the manufacturer's instructions. The DNA was processed for bisulfite modifications, which indicates the conversion of nonmethylated cytosine into uracil while 5-methylcytosine remains unmodified. The bisulfite reaction was performed according to published protocols [34], which were modified to use small quantities of DNA. Briefly, DNA in TE buffer was denatured by adding NaOH (3 M) and then incubating the solution for 30 min at 42°C. Subsequently, sodium bisulfite (3.9 M, pH 5), hydroquinone (10 mM), and nanopure H_2_O were added, and the solution was incubated at 55°C for 16 h. The resulting modified DNA was purified (Wizard® Clean-Up de Promega kit) and then eluted using nuclease-free water. The modified and purified DNA was used as a template for methylation-specific PCR (MSP) targeting the intergenic region of the RLN gene. *β*-tubulin IV was used for normalization (intergenic RLN region primers: forward, 5′-GGTGTTAAATTTTTGTAGTATTGGGGAC-3′, and reverse, 5′-TCCTTAAAATAATCC AACAACACGC-3′. *β*-tubulin IV primers: forward, 5′-GGAGAGTAATATGAATGA TTTGGTG-3′, and reverse, 5′-CATCTCCAACTTTCCCTAACCTACTTAA-3′) [35]. PCRs were performed using Go-Taq Green Master Mix® (Promega). Each reaction was amplified using the following program: one cycle at 95°C for 3 minutes for initial denaturation; 40 cycles consistent at 95°C for 15 seconds for denaturation, 58.9°C for 1 minute for annealing, and 72°C for 30 seconds for extension. After completing 40 cycles, one cycle is applied at 72°C for 5 minutes, for final extension. Finally, the samples were maintained at 16°C. The amplified products were analyzed using electrophoresis on a 2% agarose gel that was stained with Gelstar® (Cambrex Bio Science Rockland, Inc.) and then visualized under UV light. A densitometric analysis was performed using NIH Scion Image software.

### 2.5. Immunofluorescence

Naive and tetanized hippocampal rat hippocampal slices were placed in 4% PFA/4% sucrose for 30 minutes and then placed in 30% sucrose. The slices were washed 3 times with PBS, embedded in medium for frozen tissue specimens (OCT) and later sectioned at 30 *μ*m using a cryostat at −20°C. Free-floating sections were bathed in permeabilization/blocking buffer (0.7% Triton X-100 (PBS-TX), 0.1% sodium borohydride, and 10% goat serum) overnight at 4°C. The sections were later incubated with primary rabbit polyclonal antibodies against MeCP2 that had been phosphorylated at Ser-80 or at Ser-421 (dilution 1 : 200, ECM Biosciences) or with a mouse monoclonal anti-*β*-tubulin III antibody (1 : 500, Millipore) overnight at 4°C in 0.7% PBS-TX and 10% goat serum. After the sections were exposed to the primary antibodies, the sections were washed and incubated for two hours with donkey-anti-rabbit Alexa Fluor 546, donkey-Alexa Fluor 488 anti-rabbit (1 : 200), or donkey Alexa Fluor 488 anti-mouse (1 : 500) antibodies, depending on the primary antibody that was used. All secondary antibodies were obtained from Molecular Probes. Nuclei were stained using Hoechst® 33342 according to the manufacturer's instructions (Molecular Probes). Images were obtained using a confocal microscope (Nikon Eclipse C180i) with 3 laser excitation lines and the following respective emission filters: 408 nm (450/35), 488 nm (515/30), and 543 nm (605/75). Fluorescence intensity was measured using the NIS-Elements software viewer 4.0 and the EZ-c1 3.90 free viewer.

### 2.6. Statistical Analysis

The one-sample Mann-Whitney test was used to assess changes in the methylation state and expression of RLN in the hippocampus and Student's *t*-test for analysis of MeCP2 phosphorylation.

## 3. Results

**Figure 1:**
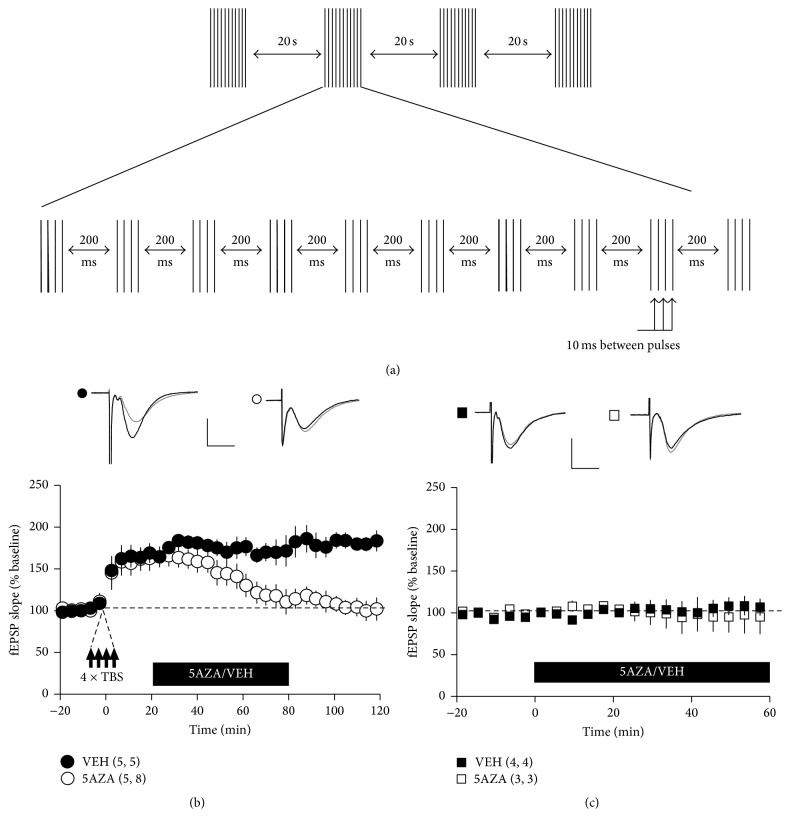
Early inhibition/capturing of DNMT impairs LTP maintenance. (a) L-LTP was induced using theta burst stimulation (TBS), which consisted of four theta epochs delivered at 0.1 Hz. Each epoch, in turn, consisted of 10 trains of four pulses (at 100 Hz) that were delivered at 5 Hz. (b) The field excitatory postsynaptic potential (fEPSP) slope was normalized to the average value during the 20 min before TBS in each experiment. Hippocampal slices were exposed to a 5AZA or vehicle (gray bar) 20 min after tetanization. Less L-LTP was induced by TBS in slices that were treated with 5AZA (25 *μ*M; open circles) than in slices treated with vehicle (0.001% CH3COOH; closed circles). Inset: representative fEPSPs are shown before (gray line) and at 120 min after (black line) LTP induction. (c) Hippocampal slices were exposed to either a 5AZA or vehicle (gray bar). In the absence of LTP induction, synaptic efficacy was not affected by either 5AZA (25 *μ*M; open square) or vehicle (0.001% CH3COOH; closed square). Inset: representative traces are shown before (gray line) and at 60 min after (black line) initial exposure to 5AZA or vehicle. For all panels, the calibration bar indicates 1 mV and 5 ms; the error bars indicate the SE and numbers in parentheses corresponds to the number of animals and number of slices.

A previous study showed that preexposing slices to DNMTs capturers/inhibitors (e.g., 5AZA and Zebularine) for 20 minutes before the induction of LTP resulted in an immediate and significant reduction in both the induction and the expression of L-LTP, suggesting that DNMTs play an important role in both DNA methylation and synaptic plasticity [7]. To more specifically test the effect of blocking/capturing DNMT during LTP, we incubated slices with 5AZA (30 *μ*M) twenty minutes after TBS to avoid disrupting LTP induction. Interestingly, exposing tetanized slices to 5AZA resulted in significantly less LTP than those observed in the slices treated with vehicle and near baseline values at 1 h after drug application (Figure 1(b), white circles) without a significant influence on basal synaptic transmission in the absence of TBS (Figure 1(c)).

**Figure 2:**
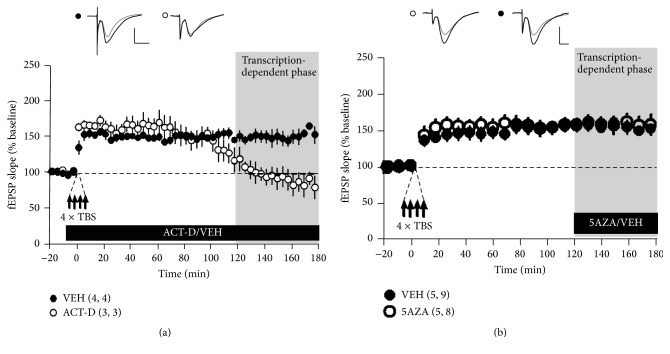
Late inhibition/capturing of DNMT has no effect on the transcription-dependent phase of LTP. (a) Less L-LTP was induced by TBS in slices treated with actinomycin-D (25 *μ*M; open circles) than in slices treated with vehicle (0.01% DMSO; closed circles). Inset: representative traces are shown 2 min before (gray line) and 180 min after (black line) LTP induction. The calibration bar indicates 1 mV and 5 ms. (b) Hippocampal slices were exposed to a 5AZA or vehicle (gray bar) at 120 min after tetanization. The amount of L-LTP that was induced by TBS was not affected in the slices treated with 5AZA (25 *μ*M; open circles) compared to the slices treated with vehicle (0.001% CH3COOH; closed circles). Inset: representative traces are shown before (gray line) and at 180 min after (black line) LTP induction. For all panels, the calibration bar indicates 1 mV and 5 ms; the error bars indicate the SE and numbers in parentheses correspond to the number of animals and number of slices.

Given that L-LTP involves the activity-dependent regulation of gene expression [36–38], we studied the effects of DNA methylation during the period when L-LTP is sensitive to inhibitors of transcription. In agreement with previous studies [38, 39], we found that blocking gene transcription using actinomycin-D (25 *μ*M) impaired L-LTP without attenuating E-LTP and that synaptic transmission returned to baseline values at 2-3 h after tetanization (Figure 2(a)). In contrast, in the vehicle-treated slices, LTP was maintained for three hours.

Surprisingly, superfusing slices with 5AZA two hours after TBS had no effect on LTP (Figure 2(b)), suggesting that L-LTP is modulated by DNA methylation only during its early phases.

### 3.1. Theta Burst Stimulation Reduces DNA Methylation on the RLN Gene

**Figure 3:**
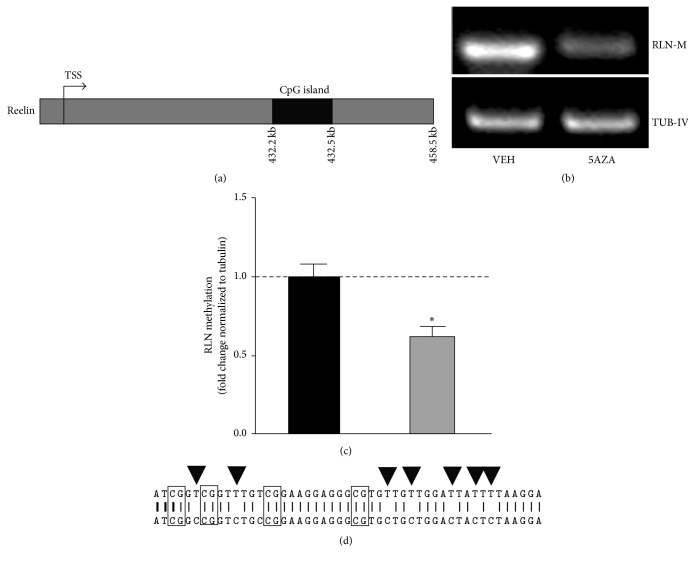
DNA methylation in the intergenic CpG island of the RLN gene. (a) Schematic representation of the location of the CpG island (solid box) relative to the transcription initiation site (TSS) [7]. (b) Hippocampal slices were exposed to 5AZA (25 *μ*M) for one hour. Microdissected CA1 tissue was processed immediately after treatment. Representative agarose gel showing that the amount of methylated DNA was lower in the 5AZA-treated slices than in the vehicle-treated slices. (c) Quantification of the methylated RLN gene normalized to the level of tubulin in data such as that shown in (b). ^*∗*^ *p* < 0.05, *n* = 5, one-sample Mann-Whitney test. (d) Bisulfite-modified DNA sequencing of the RL gene. The bisulfite-modified DNA sequence (upper) and the unmodified genomic DNA sequence (lower) were compared. All potentially methylated CG sites are labeled with a black box. When the template was unmethylated, the cytosine residues in the bisulfite-modified DNA sequence (black arrowheads) were converted from C to T.

The activation of gene transcription is associated with the loss of DNA methylation at regulatory sequences [4, 40, 41]. We therefore expected that, within the two hours during which DMNT inhibition/capturing was able to block LTP, changes in DNA methylation influenced gene transcription. To test this hypothesis, we analyzed the methylation state of the intergenic region of RLN gene. This area of the gene is required for its neuronal activity-dependent transcription (Figure 3(a)).

In replicating cells, 5AZA forms an irreversible complex DNA-DNMT, which captures DNMT in the genome, which in turn inhibits DNA methylation. 5AZA is one of the azanucleosides inhibitors with the highest potency and effectiveness, used in clinical trials approved by the FDA for the treatment of myelodysplastic syndrome [42, 43]. The low effectiveness of SAM competitive inhibitor compared with azanucleoside inhibitors was our reason for choosing 5AZA to be used in our study, which despite the potential cytotoxic effects causes DNA demethylation in neurons through a mechanism that is not yet fully established.

To determine how reliable our detection was in the MSP analysis, we first characterized changes in the methylation status of the RLN gene in adult hippocampus slices that were treated with 5AZA.

Microdissected CA1 tissues showed a robust decrease in methylated DNA in response to inhibition with DNMT (Figures 3(b) and 3(c)). We also tested the specificity of our procedure by sequencing the amplified PCR product (upper sequence) and comparing it to the NCBI database RLN gene (lower sequence). This gave us an identity of 86% (Figure 3(d)), a value that can be explained by the bisulfite modifications of unmethylated cytosines (Figure 3(d), black arrowheads). This activity was prevented at methylated cytosines in specific CpGs (Figure 3(d), black boxes).

**Figure 4:**
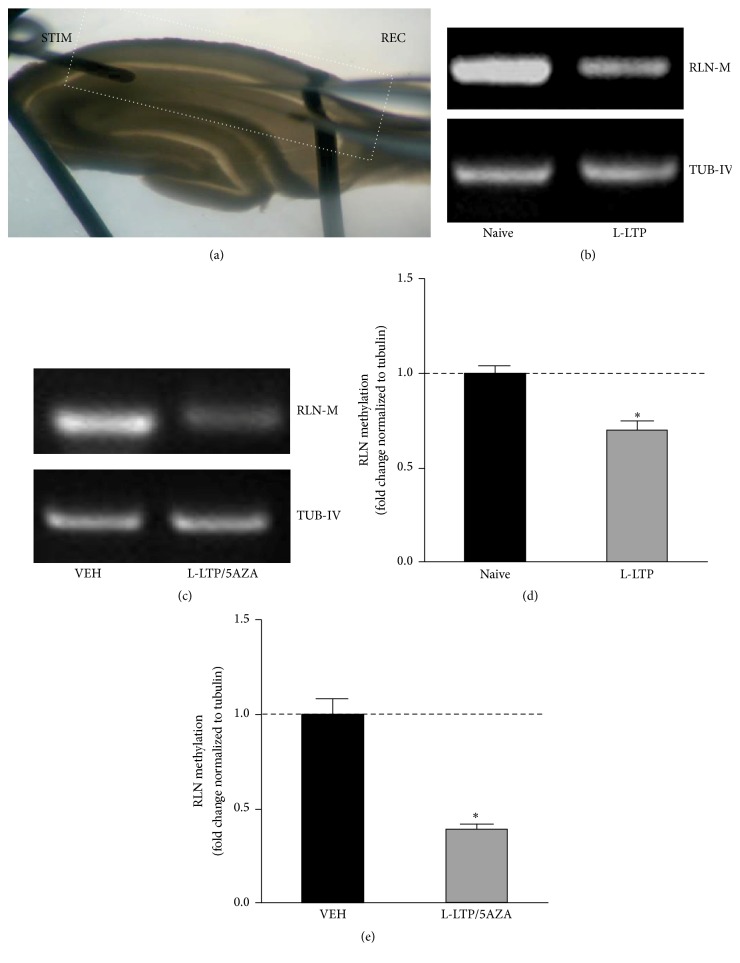
The RLN gene was methylated two hours after TBS in both the absence and the presence of 5AZA. (a) A hippocampal section showing the placement of the stimulation electrode (STIM) and the recording electrode (REC) in the stratum radiatum layer of CA1. The white dotted line represents the area that was microdissected for MSP analysis. (b) Representative agarose gel electrophoresis showing the amount of DNA that was methylated in the tetanized slices relative to the level in the naive slices at two hours after TBS. (c) Quantification of the methylation of the RLN gene normalized to the level of tubulin for data such as that shown in (c). ^*∗*^ *p* < 0.05, *n* = 5, one-sample Mann-Whitney test. (d) Representative agarose gel electrophoresis showing methylated DNA levels in tetanized slices relative to the level in the vehicle at two hours after TBS in tissues incubated in the presence of 5AZA. (e) Quantification of the level of RLN gene methylation normalized to the level of tubulin methylation for data such as that shown in (c). ^*∗*^ *p* < 0.05, *n* = 3, one-sample Mann-Whitney test.

We next investigated whether RLN undergoes acute changes in its methylation status in response to TBS-induced hippocampal LTP. A significantly lower amount of methylated DNA was observed in microdissected CA1 tissues obtained from slices in which a robust LTP was induced (>2 h, Figure 4(a)) than in naive slices (Figures 4(b) and 4(d), naive = 1.0 ± 0.046; L-LTP = 0.698 ± 0.048; *n* = 5 animals, *p* < 0.05, Mann-Whitney test). In combination with the decrease in the DNA methylation of the RLN gene following TBS-induced LTP, there were also more RLN mRNA transcripts in the treated slices than in the naive slices (data not shown). These results demonstrate that the expression of L-LTP involves changes in the methylation of the RLN gene and a correlated increase in its transcription.

As expected, blocking/capturing DMNT using 5AZA twenty minutes after LTP induction resulted in the methylation of the analyzed gene being significantly reduced to a level that was lower than was observed in the tetanized slices (Figures 4(c) and 4(e); VEH = 1.0 ± 0.081; L-LTP/5AZA = 0.3945 ± 0.0279, *n* = 3 animals; *p* < 0.05; Mann-Whitney test).

### 3.2. Dynamic Changes on MeCP2 Phosphorylation

To understand how neuronal activity can influence the transcription level of genes involved in LTP expression through DNA methylation, we studied the levels at which MeCP2 was phosphorylated at its serine 80 (MeCP2-S80) and serine 421 (MeCP2-S421) residues, both of which are known to be controlled by neuronal activity and to regulate its binding to methylated and unmethylated regions in the genome [44].

**Figure 5:**
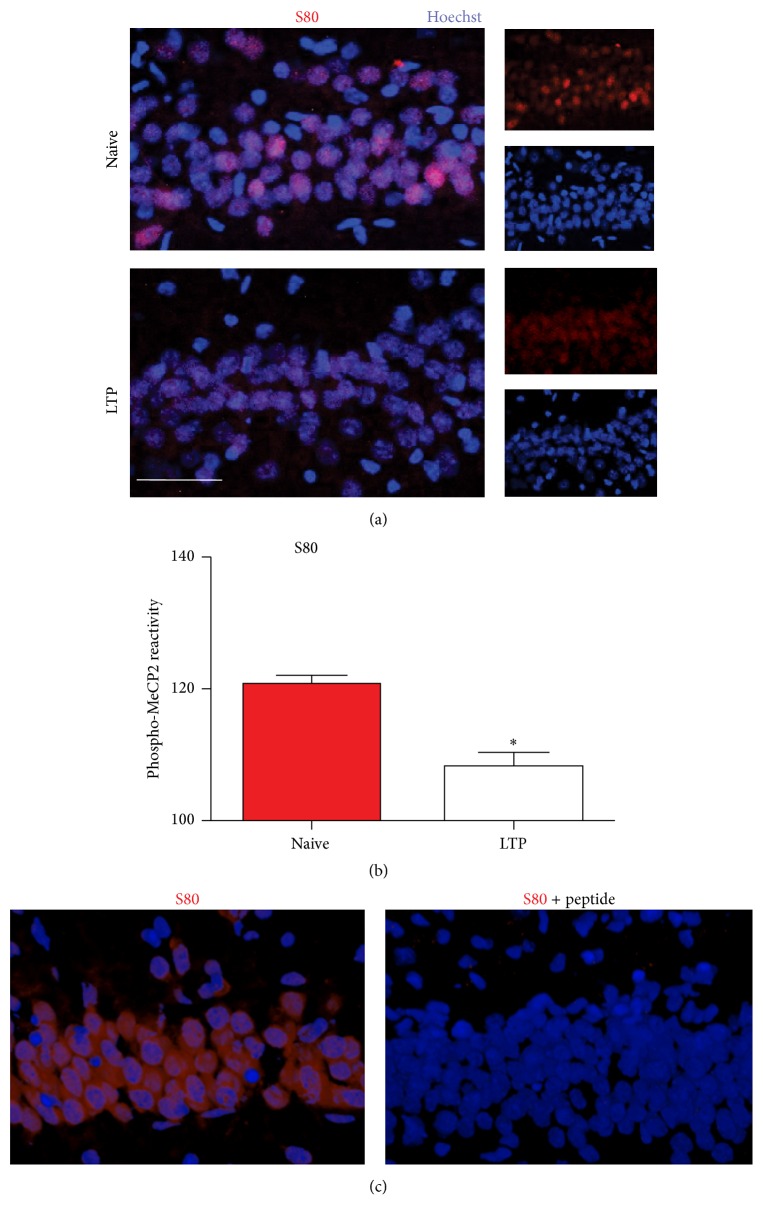
The phosphorylation of MeCP2 at S80 at two hours after tetanic stimulation. (a) Representative immunostaining for phospho-S80 MeCP2 (red) and nuclear marker Hoechst 33342 (blue) in the hippocampal CA1 region in naïve (number of nuclei = 198) and tetanized (number of nuclei = 173) slices at two hours after TBS. Calibration bar, 50 *μ*m. (b) Quantification of phospho-MeCP2 reactivity for data such as that shown in (a). ^*∗*^ *p* < 0.05, using Student's *t*-test. (c) Immunostaining for phospho-S80 MeCP2 (red) and the neuronal marker Hoechst 33342 (blue) in naïve slices incubated in the absence (left panel) or presence of treatment with an immunogen peptide (right panel) to which the relevant antibody was generated.

**Figure 6:**
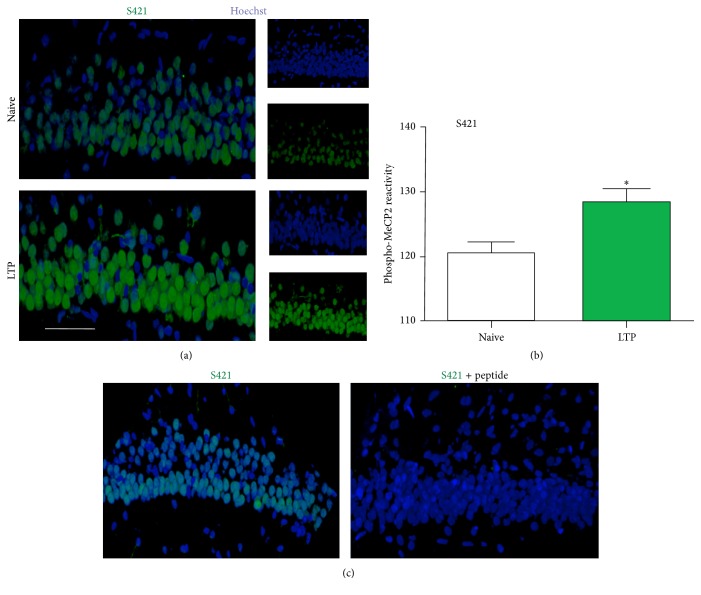
The phosphorylation of MeCP2 at S421 at two hours after tetanic stimulation. (a) Representative immunostaining for phospho-S421 MeCP2 (green) and the nuclear marker Hoechst 33342 (blue) in the hippocampal CA1 region in naïve (number of nuclei = 136) and tetanized (number of nuclei = 164) slices at two hours after TBS. Calibration bar, 50 *μ*m. (b) Quantification of phopho-MeCP2 reactivity for data such as that shown in (a). ^*∗*^ *p* < 0.05, using Student's *t*-test. (c) Immunostaining for phospho-S421 MeCP2 (green) and the neuronal marker Hoechst 33342 (blue) in the hippocampal CA1 region in tetanized slices at two hours after TBS in the absence (right panel) or presence of treatment with the immunogen peptide (left panel) to which the antibody was generated.

**Figure 7:**
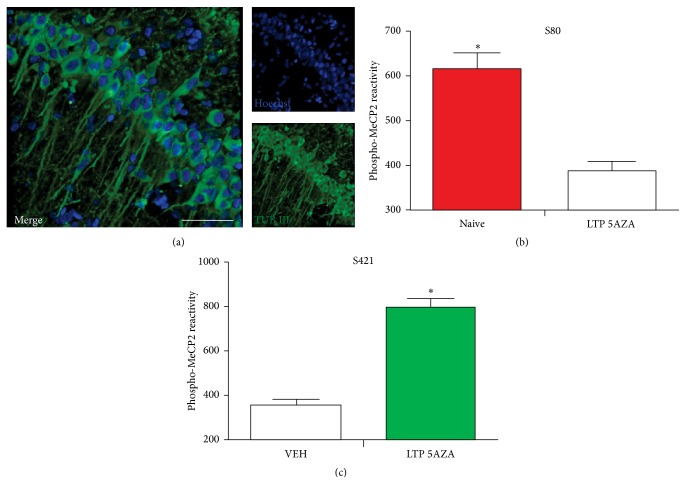
The phosphorylation of MeCP2 at S80 and S421 at two hours after tetanic stimulation in the presence of 5AZA. (a) Representative immunostaining for the neuronal marker anti-*β*-tubulin III (green) and Hoechst 33342 (blue) in the hippocampal CA1 region. Calibration bar, 50 *μ*m. (b) Quantification of the phosphorylation of MeCP2 at S80 at vehicle (number of nuclei = 62) and tetanized (number of nuclei = 98) slices at two hours after tetanic stimulation in the presence of 5AZA. ^*∗*^ *p* < 0.05, using Student's *t*-test. (c) Quantification of the phosphorylation of MeCP2 at S421 at vehicle (number of nuclei = 136) and tetanized (number of nuclei = 164) slices at two hours after tetanic stimulation in the presence of 5AZA. ^*∗*^ *p* < 0.05, using Student's *t*-test.

Slices were exposed to different experimental conditions and then tested with antibodies that specifically recognize the phosphorylated residues at MeCP2-S80 or MeCP2-S421. Immunoreactivity for MeCP2-S80 was weaker in tetanized slices, which showed stable L-LTP that lasted over two hours, than in naive slices (Figures 5(a) and 5(b)), while MeCP2-S421 reactivity was stronger (Figures 6(a) and 6(b)). Colocalization with Hoechst nuclear stain showed that antibody reactivity was limited to the nuclear region (Figures 5(a), 5(c), 6(a), and 6(c)). Incubating slices from different experimental groups with high concentrations of the immunogenic peptide to which the appropriate antibody was raised resulted in a strong decrease in fluorescence to barely detectable levels (Figures 5(c) and 6(c)). These results demonstrate the specificity of the detection method. Finally, to show that the fluorescent nuclear profiles of the MeCP2-S80 and MeCP2-S421 antibodies were not due to an unspecific somatic signal, we compared the profiles to patterns that were observed when we used an antibody raised against *β*-tubulin III. These data showed that the MeCP2 protein was in all cases confined to the nucleus (Figure 7(a)).

Because 5AZA is able to modify the expression of MeCP2 [45] and because DNA methylation is a phenomenon that is closely associated with the ability of MeCP2 to recognize these changes, we next assessed whether the presence of 5AZA alters LTP- induced phosphorylation patterns. We found that, at two hours after the L-LTP induction protocol was applied, the level of immunoreactivity for S80 was lower in the tetanized slices incubated in the presence of 5AZA (Figure 7(b)), while the level of immunoreactivity for S421 was higher (Figure 7(c)) than in the vehicle-exposed slices that were not treated with 5AZA.

## 4. Discussion

### 4.1. Inhibiting/Capturing DNMTs Impairs Hippocampal L-LTP Only during a Limited Time Window

In this study, we shed light on the dynamic process through which gene expression is controlled by DNA methylation during synaptic plasticity.

The early phases of LTP (E-LTP) do not require gene transcription. However, previous studies have shown that inhibiting/capturing DNMT prior to the induction of LTP has a robust effect on E-LTP [7]. Consistent with these findings, our results show that exposure to 5AZA twenty minutes after tetanization (to avoid interfering with the induction and early phases of LTP) resulted in significantly less LTP (Figure 1(b)). These results suggest that methylation affects LTP by modulating very early transcriptional processes. Recent studies have shown that dynamic methylation/demethylation cycles are involved in the transcriptional regulation of the trefoil factor 1 gene by o-estrogens in MCF-7 human cells [46, 47]. These data indicate that cyclical changes in the DNA methylation status of a gene can be a critical component of the complex machinery that controls its transcription and could be an active participant as a mechanism for activity-induced plasticity.

Our data demonstrate that there is a critical time window during which DNA methylation processes can affect LTP maintenance. This window is temporally correlated with the time during which gene transcription is required for the late phase of LTP [48]. These studies suggest that electrical stimulation protocols that induce plasticity activate a complex mechanism that regulates DNA methylation, which can be disturbed only during the early stages of the process, before transcriptional dependency.

### 4.2. Inhibiting/Capturing DNMT Decreases the DNA Methylation of the RLN Gene

We have shown using hippocampal slices that the methylation of the RLN gene decreases in response to tetanic stimulation, an effect that persists for at least two hours after the induction of LTP. Consistent with our results, pharmacological LTP induced with phorbol ester resulted in rapid demethylation of the RLN promoter [7]. Newer genome-wide methods for analyzing DNA methylation status have revealed that neuronal activity induces rapid and active DNA modifications in brain genes that are associated with synaptic plasticity in vivo [49].

Interestingly, slices superfused with 5AZA twenty minutes after the induction of LTP exhibited even less methylation than the slices incubated in the absence of an inhibitor of DNMTs, suggesting that there is a synergistic effect between inhibitors of DNMT and tetanic stimulation.

Consistent with our data, inhibiting/capturing DNMT1 blocked hippocampus-dependent memory formation in a contextual fear-conditioning paradigm [35]. Moreover, one hour after training, animals showed significantly less RLN gene methylation than the controls and returned to baseline within 24 h of training [35].

Altogether, these data suggest that the aberrant DNA methylation of critical genes may explain why LTP is lost in tissues incubated with 5AZA. Moreover, these data highlight the highly sensitive nature of DNA methylation processes during the early stages of LTP maintenance. In fact, evidence indicates that perturbing epigenetic regulatory mechanisms can have devastating effects on neuronal functions [50].

Compelling evidences have shown that DNA methylation in neurons appears to be governed by different rules than other cell types, as has been suggested by other authors [8]. Important evidence supporting an unusual methylation mechanism in neurons is that brain exhibits high levels of 5-hydroxymethylation [51], a modification that leads to DNA demethylation in the absence of cell division [20].

### 4.3. Inhibiting/Capturing DNMT Has No Effect on the MeCP2 Phosphorylation Induced by Tetanic Stimulation

We found that, two hours after the application of tetanic stimulation, the S421 residue of MeCP2 was phosphorylated, while the S80 residue was less phosphorylated compared to a resting condition. Furthermore, 5AZA does not affect phosphorylation patterns in tetanized slices, suggesting that the mechanisms affected by the inhibitor are not related to changes in its phosphorylation.

Different forms of synaptic plasticity might be explained, at least in part, as interplay between calcium-dependent phosphorylation and dephosphorylation events [52, 53]. Since the first report that showed that neuronal depolarization resulted in the calcium-dependent phosphorylation of MeCP2 and its subsequent release from regulatory regions of genes such as Bdnf [24], remarkable progress has been made in exploring the roles of posttranslational modifications of MeCP2, some of which activate or inhibit transcription [21].

In particular, we studied the phosphorylation of S421, which is selectively expressed in neuronal tissues [3] and is modified by calcium influx and the subsequent activation of calcium/calmodulin-dependent protein kinase IV [44, 53].

Consistent with our data, a recent study showed that a hippocampal-dependent behavioral task increased the phosphorylation of S421 [25]. Although it was thought that the phosphorylation of S421 was related only to its selective detachment to DNA, more detailed genomic distribution analyses of phospho-S421 have revealed that, under both resting and stimulated conditions, MeCP2 is not released from the target sequences in the DNA. Therefore, the additional phosphorylation events that have been described for MeCP2 must necessarily also involve the regulation of DNA binding because neural activity modifies other residues on MeCP2.

Because it has been shown that the dephosphorylation of S80 does not necessarily coincide with the phosphorylation of S421 or vice versa [53], we studied the effect of the S80 residue, which is the most constitutively phosphorylated residue in resting neurons and is dephosphorylated by neuronal activity [44, 53]. In contrast to S421, we found that tetanic stimulation also activates unidentified calcium-dependent phosphatases that dephosphorylate the S80 residue and that this is a critical event during synaptic plasticity [53]. Functionally, the phosphorylation of S80 does not affect the overall subcellular localization of MeCP2, but it has a strong impact on the affinity of this protein for DNA [3, 53].

Finally, the data presented in this work raise a number of new questions that must be addressed in the future, and although the mechanisms by which the azanucleosides inhibit DNA methylation are not fully understood, its use in the future will continue [7, 35, 54] providing valuable information about DNA methylation in synaptic plasticity, learning, and memory.
